# Deep Learning for Classification and Selection of Cine CMR Images to Achieve Fully Automated Quality-Controlled CMR Analysis From Scanner to Report

**DOI:** 10.3389/fcvm.2021.742640

**Published:** 2021-10-14

**Authors:** Vittoria Vergani, Reza Razavi, Esther Puyol-Antón, Bram Ruijsink

**Affiliations:** ^1^School of Biomedical Engineering and Imaging Sciences, King's College London, London, United Kingdom; ^2^Department of Adult and Paediatric Cardiology, Guy's and St. Thomas' NHS Foundation Trust, London, United Kingdom; ^3^Department of Cardiology, Division of Heart and Lungs, University Medical Centre Utrecht, Utrecht, Netherlands

**Keywords:** cardiac magnetic resonance, deep learning, quality control, cardiac function, view-selection, multivendor

## Abstract

**Introduction:** Deep learning demonstrates great promise for automated analysis of CMR. However, existing limitations, such as insufficient quality control and selection of target acquisitions from the full CMR exam, are holding back the introduction of deep learning tools in the clinical environment. This study aimed to develop a framework for automated detection and quality-controlled selection of standard cine sequences images from clinical CMR exams, prior to analysis of cardiac function.

**Materials and Methods:** Retrospective study of 3,827 subjects that underwent CMR imaging. We used a total of 119,285 CMR acquisitions, acquired with scanners of different magnetic field strengths and from different vendors (1.5T Siemens and 1.5T and 3.0T Phillips). We developed a framework to select one good acquisition for each conventional cine class. The framework consisted of a first pre-processing step to exclude still acquisitions; two sequential convolutional neural networks (CNN), the first (CNN_class_) to classify acquisitions in standard cine views (2/3/4-chamber and short axis), the second (CNN_QC_) to classify acquisitions according to image quality and orientation; a final algorithm to select one good acquisition of each class. For each CNN component, 7 state-of-the-art architectures were trained for 200 epochs, with cross entropy loss and data augmentation. Data were divided into 80% for training, 10% for validation, and 10% for testing.

**Results:** CNN_class_ selected cine CMR acquisitions with accuracy ranging from 0.989 to 0.998. Accuracy of CNN_QC_ reached 0.861 for 2-chamber, 0.806 for 3-chamber, and 0.859 for 4-chamber. The complete framework was presented with 379 new full CMR studies, not used for CNN training/validation/testing, and selected one good 2-, 3-, and 4-chamber acquisition from each study with sensitivity to detect erroneous cases of 89.7, 93.2, and 93.9%, respectively.

**Conclusions:** We developed an accurate quality-controlled framework for automated selection of cine acquisitions prior to image analysis. This framework is robust and generalizable as it was developed on multivendor data and could be used at the beginning of a pipeline for automated cine CMR analysis to obtain full automatization from scanner to report.

## Introduction

Cardiac magnetic resonance (CMR) is the state-of-the-art clinical tool to assess cardiac morphology, function, and tissue characterization (1), and both European and American guidelines advocate its use to diagnose and monitor a large number of cardiovascular diseases (2, 3). The role of CMR continues to grow due to the technical advances that allow increasingly detailed analysis of the cardiovascular system.

However, systematic manual analysis of the different CMR sequences which are acquired during a typical CMR exam is highly time consuming, where the bulk of the time is taken up by repetitive tasks, such as image identification, selection, and segmentation, which are at the basis of CMR post-processing.

Deep learning (DL), a branch of artificial intelligence (AI), is securing an emergent role in the field of CMR, as it provides for automatization of repetitive tasks, significantly reducing the time required for image analysis, while maintaining a high degree of accuracy (4, 5). Physicians' time can thus be optimized and targeted for critical review of clinical and imaging information to reach a correct diagnosis. Moreover, automated analysis allows access to biomarkers of cardiac function that would normally be too labor intensive to obtain, such as peak ejection and filling rates from ventricular volume curves (6, 7) or atrioventricular valve planar motion (8) from long-axis segmentations.

Several groups have shown promising results on the implementation of DL in the analysis of CMR, including segmentation of cine images to derive cardiac function (5), analysis of perfusion defects to detect inducible ischemia (9), and assessment of late gadolinium enhancement and T1 mapping to aid tissue characterization (10, 11).

However, some limitations still need to be addressed before a widespread clinical adoption of DL tools, such as steps to perform automated selection of target images from the full CMR exam, as well as robust systems to flag inadequate quality of image or of analysis, which are the necessary steps that precede analysis of CMR sequences in the clinical setting. We have previously shown that comprehensive quality-control can be introduced into a DL pipeline for accurate, automated analysis of cine CMR images that adheres to clinical safety standards (5). On the other hand, DL has not yet been systematically implemented for image recognition and selection prior to analysis in CMR.

This study aimed at developing a framework for automated identification and quality-controlled (QC) selection of cine images used for cardiac function analysis from routine clinical CMR exams. This framework was then implemented as the first step of a larger pipeline for QC CMR analysis of cine images previously developed by our group (5).

## Materials and Methods

**Figure 1:**
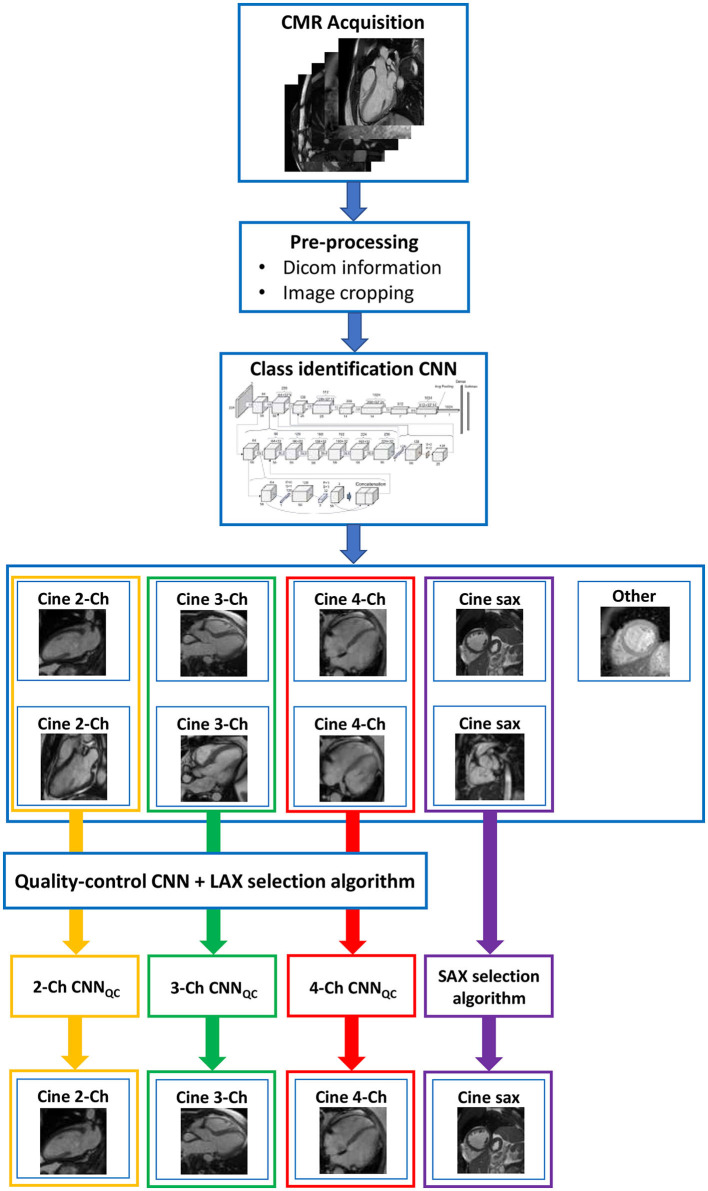
Complete framework. The framework consisted of a first pre-processing step to exclude still images; two sequential convolutional neural networks (CNN), the first to classify images in standard cine views (2/3/4-chamber and short axis), the second to classify images according to image quality and orientation; a final algorithm to select one good image of each class. This construction allows for the framework, presented with a full CMR exam, to perform a quality-controlled selection of one good image for each conventional cine class, which is then used for analysis of cardiac function. Ch, chamber; CNN, convolutional neural network; LAX, long axis; SAX, short axis; QC, quality control.

The framework we present is composed of a set of algorithms combined with two convolutional neural networks (CNN) aimed at identifying conventional cine views (CNN_class_) and at sorting these images according to quality into “correct” and “wrong” (CNN_QC_). This construction allows for the framework, presented with a full CMR exam, to perform a selection of one good quality acquisition for each conventional cine class, which is then used for analysis of cardiac function, and to flag exams when no image of sufficient quality could be identified (see Figure 1).

The developed pipeline was implemented in Python using standard libraries such as Numpy and Pandas as a dedicated deep learning library Pytorch.

### Study Population

This is a retrospective multivendor study conducted on a large CMR dataset. 3,445 CMR exams were included: 1,510 from UK Biobank and 1,935 from Guy's and St. Thomas' NHS Foundation Trust (GSTFT), London, comprising of a total of 119,285 individual CMR acquisitions. Images were acquired on 1.5T Siemens and 1.5T and 3.0T Phillips CMR scanners using a large variety of protocols, with variable voxel- and image-size, acquisition techniques and under-sampling factors.

Our dataset was randomly selected from the pool of available studies in the UK Biobank and GSTFT databases. All exams were acquired between 2004 and 2020. The random selection was used to obtain a heterogeneous population, including both healthy and pathological hearts, with a variety of cardiac pathologies (ischemic heart disease, dilated and hypertrophic cardiomyopathy, valvular heart disease, adult congenital heart disease (ACHD), and others). In the case of grossly disruptive artifacts (e.g., device artifacts covering the majority of the chambers), or grossly distorted anatomy (e.g., patients with single-ventricle morphology or Ebstein's disease), CMR exams were excluded from the dataset.

### Identification of Conventional Cine Classes

The first part of our framework aimed to identify the standard cine views used for analysis of cardiac function from the complete exam. These were the 2-Chamber view (2Ch), 3-Chamber view (3Ch), 4-Chamber view (4Ch), and the short-axis stack (SAX).

First, all single-frame acquisitions were excluded from the exams based on the dicom-header information. This allowed to reduce the bulk of acquisitions available in a complete CMR exam to a set of multi-frame acquisitions. The 2Ch, 3Ch, 4Ch, and SAX sequences were subsequently selected from the remaining acquisitions using our view selection algorithm. To identify the class of the cine acquisitions, only a single frame of the acquisition is needed. Therefore, the first frames of each of the remaining data was used for training. All data was manually classified into the conventional cine classes by an expert physician (2,905 2Ch, 1,171 3Ch, 2,963 4Ch, 9,112 SAX images) and a class of “other” (4,043). In case of doubt, a second opinion was sought, and a decision was made by consensus. Prior to CNN training, all images were cropped to a standard size of 256 × 256 pixels and converted to numpy arrays.

The manually classified data were divided as follows: 80% was used for training, 10% was used for validation, and 10% for testing. The training, validation and test data cohorts had a mutually exclusive subject pool, i.e., acquisitions from the same subject could only be used in one of the three cohorts. We trained seven state-of-the-art CNN architectures: AlexNet (12), DenseNet (13), MobileNet (14), ResNet (15), ShuffleNet (16), SqueezeNet (17), and VGG (18). Each network was trained for 200 epochs with cross entropy loss, to classify end-diastolic images into the five classes described. For training data, data augmentation was performed on-the-fly using random translations (±30 pixels), rotations (±90°), flips (50% probability) and scalings (up to 20%) to each mini-batch of images before feeding them to the network. The probability of augmentation for each of the parameters was 50%. Augmentation is the only technique we use to prevent over-fitting, as other techniques were not found to improve performance and their omission contributed to a simpler network architecture.

An additional algorithm was used after CNN_class_ to check complete classification of the short axis acquisition. An image classified as SAX was confirmed to belong to a short axis acquisition if the following two criteria, screened by the algorithm, were met: (1) the image belonged to a stack composed of a minimum of 8 slices, (2) at least 2 out the 3 central images of the stack were classified as short axis by CNN_class_.

### Quality Control of Selected Images

The second part of our framework aimed to scrutinize the quality of the identified long-axis cine images. QC of short axis acquisitions was not performed in this step, as our downstream pipeline for automated CMR analysis already includes short-axis QC (5).

To train the networks for QC (CNN_QC_), a set of 2Ch (1,937), 3Ch (1,591), 4Ch (2,003) images from our database were reviewed by an expert physician and classified as “correct” or “wrong” based on whether image quality was satisfactory for subsequent analysis. Images that included mis-triggering, breathing, implant or fold-over artifacts were classified as “wrong” if the detection of myocardial borders was hindered. Moreover, all off-axis orientations (i.e., presence of left ventricular outflow tract (LVOT) in 4Ch, fore-shortening of the apex, absence of any of the valves in 3Ch) were also classified as “wrong.” In case of doubt, a second opinion was sought, and a decision was made by consensus. The resulting database consisted of 1,444 “correct” and 493 “wrong” 2Ch, 1,098 “correct” and 493 “wrong” 3Ch images and 1,393 “correct” and 610 “wrong” 4Ch images.

The manually classified data were used to train QC networks for each class (2Ch-CNN_QC_, 3Ch-CNN_QC_, 4Ch-CNN_QC_). The data were divided as follows: 80% training, 10% for validation, 10% for testing. We trained the same seven CNN architectures as described in the previous section. We used the same training process as described for training of CNN_class_, with the difference that CNN_QC_ was trained as binary classifiers, i.e., a two-class classification problem as opposed to five, and therefore used binary cross entropy with a logit loss function. Additionally, we implemented an adaptive learning rate scheduler, which decreases the learning rate by a constant factor of 0.1 after 5 epochs stopping on plateau on the validation/test set (commonly known as ReduceLRonPlateau). This step was added as it improves CNN training when presented with unbalanced datasets. The CNN_QC_'s output was a binary classification (“correct” vs. “wrong”), as well as the probabilities (i.e., certainty) associated with the classification for each case.

### Complete Framework: From Full Study to Selection

To complete the framework, the CNN_class_ and CNN_QC_ were combined with a final selection algorithm. This algorithm selected one good quality acquisition of each standard cine view for image analysis, when multiple acquisitions of a single class were present in the exam. For long axis data, it did so by identifying the case with the highest probability of being scored “correct” by the CNN_QC_. For short axis, the stack with the highest probability of belonging to SAX (obtained from the output of the CNN_class_) was selected. If any of the classes was absent in an exam, or the framework did not identify an image of sufficient quality, the case was flagged for clinician review.

As the individual components act in series in the complete framework, their sequential action will yield an overall performance that is different from the sum of the individual ones. To find the best combination of components, each possible combination of the trained CNN architectures trained in the previous steps was tested using an additional test-set of 379 scans randomly selected from the database, not previously used for CNN training. For each exam, a manual operator selected the best cine long and short axis acquisitions. To determine the intra- and inter-observer variability present in the manual analysis, 100 randomly selected scans were re-analyzed by the same operator and by a second operator.

### Statistics

#### Class Identification CNN

Precision, recall, and F1-score of each class (“4Ch,” “3Ch,” “2Ch,” “SAX,” “other”) and overall accuracy were computed at test-time to evaluate the performance of each trained CNN_class_.

#### Quality Control CNNs

Precision, recall, and F1-score of each class (“correct,” “wrong”) and overall accuracy were assessed to evaluate the performance at test-time of each trained 2Ch/3Ch/4Ch-CNN_QC_.

#### Complete Framework

Sensitivity (defined as: the percentage of incorrect cases identified as incorrect), specificity (defined as: the percentage of correct cases identified as correct), and balanced accuracy were computed for each framework. Cohen kappa coefficient was used to assess intra- and inter-observer variability.

### Full Pipeline: From Scanner to Report

Finally, we added the complete framework as the first step of our previously validated pipeline for quality-controlled AI-based analysis of cardiac function from CMR (5). Broadly, this pipeline consists of quality-controlled image segmentation and analysis of cine images to obtain LV and RV volumes and mass, LV ejection and filling dynamics, and longitudinal, radial and circumferential strain.

We present the feasibility and importance of a fully automated multi-step QC pipeline by (1) running 700 new CMR exams cases (not earlier seen during training) through the pipeline, and (2) presenting a video (Supplementary Video 1) of the full analysis for a good-quality case. For the 700 cases we ran through the pipeline we report the average time for selection and complete cine analysis from a full CMR study, and again report sensitivity, specificity and balanced accuracy of error detection.

## Results

### Study Population

Of the 3,827 CMR exams used for this study 3,448 were used for the training and validation of CNN_class_ and CN_QC_ [1,026 acquisition of 16,151 (6.4%) were excluded because of grossly disruptive artifacts or grossly distorted anatomy]. These included patients undergoing clinical scans at GSTFT as well as subjects voluntarily enrolling onto the UK BioBank project, yielding a heterogeneous population in terms of sex (55% male) and clinical presentation (43% healthy, the remaining displaying a wide variety of cardiovascular pathologies, as shown in Table 1).

**Table 1:** Population characteristics.

		**CNN training**	**Complete framework**
Number		3,445	379
Age (years)		57 ± 16	49 ± 19
Sex (males)		1,911 (55)	228 (60)
Height (cm)		176 ± 32	171 ± 18
Weight (kg)		79 ± 18	80 ± 19
BMI (kg/m^2^)		27 ± 5	27 ± 7
Ethnicity	Caucasian	2,401 (69.7)	231 (60.9)
	Afro-Caribbean	172 (5.0)	54 (14.2)
	Asian	85 (2.5)	10 (2.6)
	Other	21 (0.6)	7 (1.8)
	Not stated	766 (22.2)	77 (20.3)
Cardiac pathology	Healthy	1,886 (54.7)	68 (17.9)
	IHD	315 (9.1)	43 (11.3)
	DCM	167 (4.8)	27 (7.1)
	HCM	77 (2.2)	16 (4.2)
	ACHD	185 (5.4)	59 (15.6)
	Valvular	133 (3.9)	37 (9.8)
	Vascular	104 (3.0)	32 (8.4)
	Arrhythmic	159 (4.6)	26 (6.9)
	Other	419 (12.2)	71 (18.7)

*Age, sex, height, weight, and cardiac pathology of subjects used for training of CNNs, framework and full pipeline validation. All continuous values are reported as mean ± standard deviation, while categorical variables are reported as number (percentage). ACHD, adult congenital heart disease (excluding valvular and vascular abnormalities); CNN, convolutional neural network; HCM, hypertrophic cardiomyopathy; IHD, ischaemic heart disease; SD, standard deviation*.

The remaining 379 CMR scans were used to test the complete framework. These were all selected from the GSTFT database to obtain a population representative of routine clinical practice. Demographic characteristics are comparable to the training population, but clinical presentation was more variable, with only 18% of patients having no cardiovascular pathology.

Population characteristics are summarized in Table 1.

### Class Identification CNN

Precision, recall, F1-score, accuracy for all CNN_class_ are presented in Table 2. All trained architectures showed excellence performance, with accuracy ranging from 0.989 to 0.998. DenseNet and ResNet reached highest accuracy, i.e., 0.988. DenseNet showed best precision, recall and F1-score for conventional cine classes: 0.998, 1.00, 0.999 for 2Ch, 1.00, 1.00, 1.00 for 3Ch, 1.00, 0.998, 1.00 for 4Ch, and 0.996, 0.999, 0.998 for SAX.

**Table 2:** Class identification CNN_class_ performance.

	**AlexNet**			**DenseNet**			**MobileNet**			**ResNet**			**ShuffleNet**			**SqueezeNet**			**VGG**		
	**Precision**	**Recall**	**F1-score**	**Precision**	**Recall**	**F1-score**	**Precision**	**Recall**	**F1-score**	**Precision**	**Recall**	**F1-score**	**Precision**	**Recall**	**F1-score**	**Precision**	**Recall**	**F1-score**	**Precision**	**Recall**	**F1-score**
2-chamber	0.998	0.993	0.996	0.998	1.000	0.999	0.997	1.000	0.998	0.998	1.000	0.999	0.993	1.000	0.997	0.986	0.991	0.989	0.993	1.000	0.997
3-chamber	0.991	0.953	0.972	1.000	1.000	1.000	1.000	0.996	0.998	0.996	0.996	0.996	1.000	0.983	0.991	0.987	0.979	0.983	0.987	0.987	0.987
4-chamber	1.000	0.990	0.995	1.000	0.998	0.999	0.990	0.995	0.992	0.998	0.995	0.997	0.997	1.000	0.998	0.987	0.992	0.989	0.993	0.997	0.995
Short axis	0.995	0.999	0.997	0.996	0.999	0.998	0.997	1.000	0.998	0.999	0.999	0.999	0.996	1.000	0.998	0.992	0.997	0.994	0.996	1.000	0.998
Other	0.973	0.985	0.979	0.998	0.990	0.994	0.996	0.984	0.990	0.994	0.995	0.994	0.996	0.984	0.990	0.984	0.969	0.976	0.995	0.978	0.986
Accuracy	**0.992**			**0.998**			**0.996**			**0.998**			**0.996**			**0.989**			**0.994**		

*Precision, recall, and F1 score for each CNN_class_-determined class, and accuracy of each trained CNN_class_*.

### Quality Control CNN

Precision, recall, F1-score, and accuracy for all CNN_QC_ are shown in Table 3. Accuracy was variable for different architectures and ranged from 0.751 to 0.861 for 2Ch, from 0.690 to 0.806 for 3Ch, and from 0.705 to 0.859 for 4Ch. Precision, recall and F1-score were consistently lower for the “wrong” class compared to the “correct” class for all trained architectures and across the 3 different chamber views.

**Table 3:** Class identification CNN_QC_ performance.

	**AlexNet**			**DenseNet**			**MobileNet**			**ResNet**			**Shufflenet**			**SqueezeNet**			**VGG**		
	**Precision**	**Recall**	**F1-score**	**precision**	**recall**	**F1-score**	**precision**	**recall**	**F1-score**	**Precision**	**recall**	**F1-score**	**precision**	**recall**	**F1-score**	**precision**	**recall**	**F1-score**	**precision**	**recall**	**F1-score**
**2Ch-CNN_QC_**																					
Correct	0.856	0.844	0.850	0.931	0.841	0.884	0.889	0.917	0.903	0.877	0.910	0.893	0.881	0.869	0.875	0.755	0.993	0.858	0.873	0.955	0.912
Wrong	0.536	0.559	0.547	0.620	0.806	0.701	0.714	0.645	0.678	0.683	0.602	0.640	0.608	0.634	0.621	0.000	0.000	0.000	0.803	0.570	0.667
Accuracy	**0.775**			**0.832**			**0.851**			**0.835**			**0.812**			**0.751**			**0.861**		
**3Ch-CNN_QC_**																					
Correct	0.788	0.795	0.792	0.815	0.923	0.866	0.850	0.873	0.861	0.830	0.868	0.849	0.880	0.800	0.838	0.690	1.000	0.816	0.824	0.873	0.848
Wrong	0.536	0.525	0.531	0.757	0.535	0.627	0.699	0.657	0.677	0.674	0.606	0.638	0.630	0.758	0.688	0.000	0.000	0.000	0.674	0.586	0.627
Accuracy	**0.712**			**0.803**			**0.806**			**0.787**			**0.786**			**0.690**			**0.784**		
**4Ch-CNN_QC_**																					
Correct	0.877	0.821	0.848	0.900	0.900	0.900	0.891	0.878	0.884	0.878	0.900	0.888	0.906	0.832	0.867	0.705	1.000	0.827	0.891	0.878	0.884
Wrong	0.630	0.726	0.675	0.761	0.761	0.761	0.719	0.744	0.731	0.745	0.701	0.722	0.664	0.795	0.724	0.000	0.000	0.000	0.719	0.744	0.731
Accuracy	**0.793**			**0.859**			**0.838**			**0.841**			**0.821**			**0.705**			**0.838**		

*Precision, recall, and F1 score for “correct” and “wrong” cases for 2Ch-CNN_QC_, 3Ch-CNN_QC_, 4Ch-CNN_QC_, and accuracy of each trained CNN_QC_*.

### Complete Framework

Sensitivity, specificity, and balanced accuracy of each constructed framework to identify and select one good quality 2Ch, one good quality 3Ch, and one good quality 4Ch image for each exam are shown in Table 4. For the sake of brevity, we present the results of one CNN_class_, i.e., DenseNet, given the very high and similar performance of all different architectures, combined with all possible CNN_QC_'s.

**Table 4:** Framework validation.

	**Framework for image identification and selection**								
	**DenseNet CNN_CLASS_+**								
	**2Ch-CNN_QC_**			**3Ch-CNN_QC_**			**4Ch-CNN_QC_**		
**Network**	**SEN**	**SPE**	**BACC**	**SEN**	**SPE**	**BACC**	**SPE**	**SEN**	**BACC**
AlexNet	79.4	89.8	84.6	84.4	85.8	85.1	70.3	90.9	80.6
DenseNet	75.0	91.1	83.0	**93.2**	**85.3**	**89.2**	88.2	90.9	89.5
MobileNet	78.1	92.4	85.2	88.9	85.9	87.4	72.2	91.9	82.1
ResNet	77.8	91.4	84.6	88.9	85.1	87.0	83.3	90.9	87.1
ShuffleNet	**89.7**	**91.5**	**90.6**	88.9	85.9	87.4	**93.9**	**89.2**	**91.6**
SqueezeNet	55.3	88.2	71.8	51.8	91.0	71.4	23.3	95.5	59.4
VGG	84.4	91.8	88.1	84.8	85.1	84.9	79.4	91.4	85.4

*SEN, Sensitivity; SPE, Specificity; and BACC, balanced accuracy of each constructed framework for image identification and selection. Best performing framework per class is highlighted in blue*.

The best performing framework was: DenseNet CNN_class_ + ShuffleNet 2Ch-CNN_QC_ (sensitivity = 89.7%, specificity = 91.5%, balanced accuracy = 90.6%), DenseNet 3Ch-CNN_QC_ (sensitivity = 93.2%, specificity = 85.3%, balanced accuracy = 89.2%), ShuffleNet 4Ch-CNN_QC_ (sensitivity = 93.9%, specificity = 89.2%, balanced accuracy = 91.6%).

Cohen's *k* for intra- and inter-observer agreement for the same manual operator and between the two different operators were 0.79 and 0.60, respectively.

### Full Pipeline: From Scanner to Report

The sensitivity, specificity and balanced accuracy of the integrated view selection and quality-controlled cardiac analysis pipelines was 96.3, 85.0, and 90.6%, respectively. Performance was also assessed for healthy and pathological cases separately. Results are summarized in Table 5.

**Table 5:** Full pipeline performance.

	**Full pipeline**		
	**BACC**	**SEN**	**SPE**
*Healthy*	92.3	96.5	88.1
*Pathological*	87.3	95.7	78.9
Global	90.6	96.3	85.0

*SEN, Sensitivity; SPE, Specificity; and BACC, balanced accuracy of full pipeline, global, and for left (LV) and right ventricle (RV), and healthy and pathological hearts*.

The average time for selection and complete cine analysis from a full CMR study was between 4 and 7 min for a clinical CMR exam.

Supplementary Video 1 portrays how implementing the new framework prior to segmentation results in good quality analysis.

## Discussion

In this study we present a DL-based framework to identify all conventional cine views from a full CMR exam, and subsequently select one image per class of good quality for further automated image analysis. To the best of our knowledge, this is the first automated framework developed for this purpose.

The framework was trained on a large database, a prerequisite to develop DL tools of good quality. It was also trained on multivendor and clinically heterogeneous data, which makes it generalizable to be implemented as the first step for other existing tools for image analysis. Moreover, the framework was developed through training and testing of 7 state-of the art CNN architectures for each step. In DL, several network variants are available, each exhibiting different strengths and weaknesses. Studies often focus on a single highly individualized network, tailored for a task through multiple trial-and-error experiments. This makes reproduction of the methods and appreciation of its performance in the context of other datasets challenging. In our work, we present the data of all trained CNN architectures, thus displaying our selection process in a reproducible, fair, and meaningful way.

Finally, we integrated the new framework as the first step of a larger pipeline we had previously developed (8), and we demonstrated that it could produce highly accurate, rapid, and fully-automated cine analysis from a complete collection of images routinely acquired during a clinical study.

### Class Identification CNN

Class identification is the first necessary step for image analysis, making algorithmic classification of standard views a fundamental step for true automatization of analysis (19). DL has been used to meet this need for automated analysis of echo images (20, 21), but not in the field of CMR.

Identification of conventional cine classes can be seen as a trivial task. Nonetheless it is time consuming, especially in long CMR studies, where a multitude of sequences are acquired. Moreover, view recognition cannot rely on the name of the sequences, as these are not replicated across groups and misnaming is common, especially when images are repeated due to insufficient quality or slight errors in view-planning, or added during acquisition. These characteristics make the problem of class identification well-suited for automation. In computer vision tasks in particular, DL has shown excellent performance (22). This is reflected by the high performance of all trained CNN_class_ architectures, which had an accuracy approaching 100%. Precision, recall and F1-scores were mostly between 0.99 and 1 for all classes.

### Quality Control CNN

The second DL component of our framework was trained to add a quality-control step to our framework by identifying images of insufficient quality or inadequate planning to inform the automated image analysis process.

**Figure 2:**
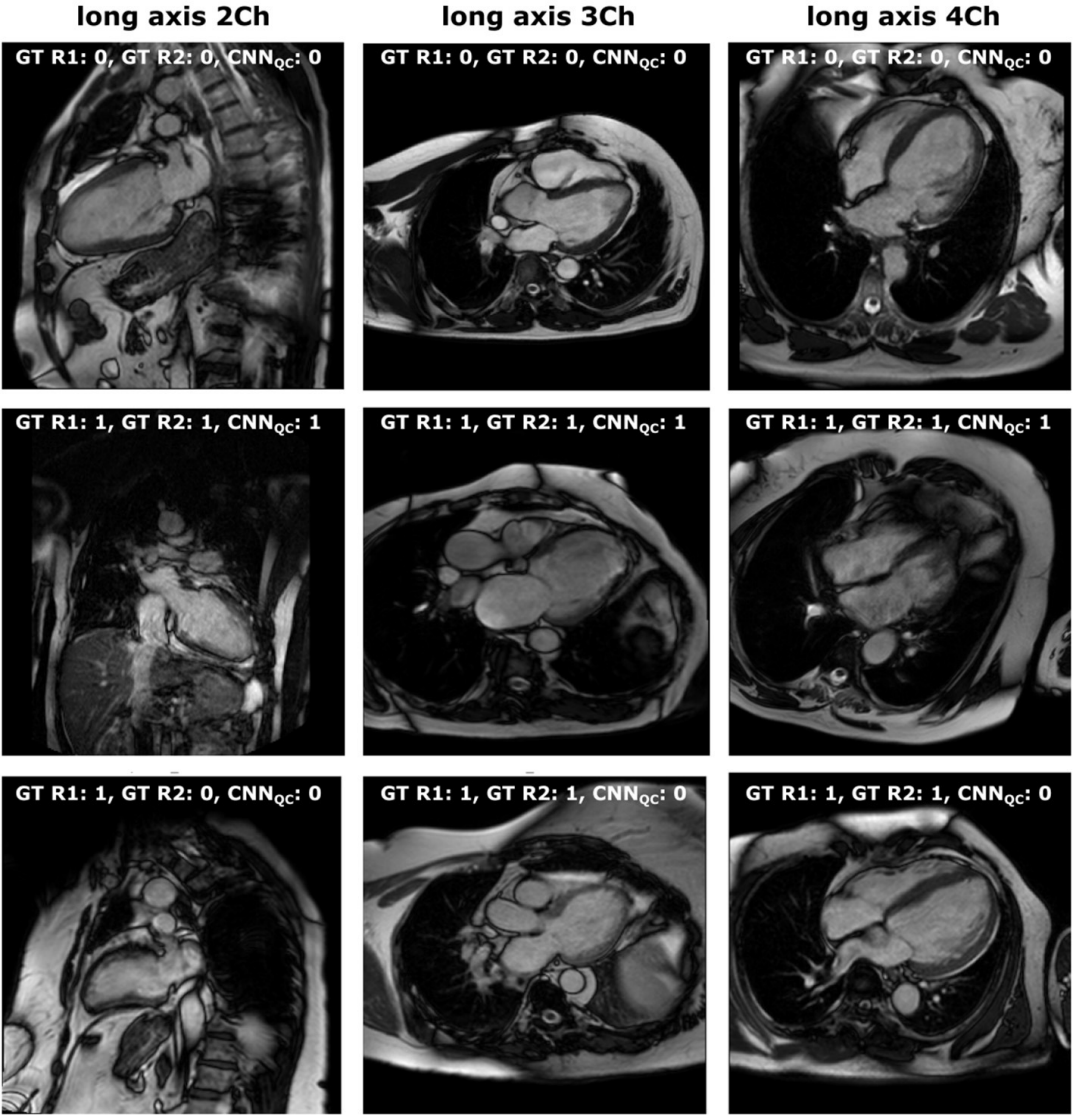
Manual vs. automated classification. Visual representation of: (first line) cases classified as “correct” both by manual assessment (both operators) and CCN_QC_; (second line) cases classified as “wrong” both by manual assessment (both operators) and CCN_QC_; (third line) cases classified as “wrong” by manual assessment (with disagreement between operators for 2-chamber) and as “correct” by CCN_QC_. 0, correct; 1, wrong; Ch, chamber; GT, ground truth; R1, first operator; R2, second operator.

Quality control is crucial to transfer DL research tools to the clinical reality in a safe manner, and its importance is increasingly recognized (5, 10, 23, 24). The performance of our CNN_QC_ was lower compared to the CNN_class_ with highest recorded accuracy of 0.86 for 2Ch and 4Ch, and 0.80 for 3Ch. This is explained by several reasons. First, the input data were highly unbalanced, which is a natural consequence of the fact that radiographers aim to acquire good quality images, resulting in the poor quality class being significantly underrepresented. This is reflected by the significantly lower precision, recall and f1-scores for the identification of “wrong” images compared to that of “correct” ones. To reduce the bias of unbalanced data, we used cross entropy loss, adaptive learning rate scheduler, and balanced accuracy, but such bias can never be fully controlled. Second, images to be considered of insufficient quality have a wide range of problems, from motion artifacts to off-axis planning of different types, making their grouping into one class difficult for the CNN. In particular, when evaluating 3Ch views, the quality of both the cardiac chambers and the aorta were considered, which might explain the lower performance compared to 2Ch and 4Ch views. On the other hand, separation into different classes would have resulted in further imbalance of the data, with insufficient numbers in each hypothetical poor-quality class. Therefore, we decided to group them together. Last, there is a degree of subjectivity in this task, as the same problem can be present to a varying degree of severity; for example, off-axis planning of the 4Ch view can result in the presence of a clear LVOT, or just a small disruption of the basal septum. The subjectivity of QC is reflected in the intra- and inter-observer variability during manual assessment. Consequently, it is both unlikely and unnecessary for any algorithm to reach 100% accuracy in this task. The cases with low degree of severity were the most likely to be misclassified, as well as the most frequent source of inter- and intra-observer disagreement, as displayed in Figure 2.

### Complete Framework

For the complete framework, we selected the CNN_class_ and CNN_QC_ that performed best in combination with a selection algorithm, rather than the networks that performed best in the validation of the individual steps. This was done for two reasons. First, a sequential process can leverage individual strengths and weaknesses to obtain the best combined result. Second, the addition of the selection algorithm after the two CNNs aimed at achieving a more complex and possibly more clinically relevant task: selection of images for further analysis, which can be highly time-consuming, especially in long exams containing several acquisitions. It was therefore our intent to test the integrated framework and select the one with the best overall performance.

The integration of the three steps of the framework yielded an accurate and rapid system to select images of interest for analysis. The best combination was DenseNet CNN_class_, ShuffleNet 2Ch-CNN_QC_, DenseNet 2Ch-CNN_QC_, ShuffleNet 4Ch-CNN_QC_, which had a 90% sensitivity for 2Ch, 93% for 3Ch, and 94% for 4Ch acquisitions. This is achieved at the cost of a small proportion of good quality images being mistakenly labeled as erroneous, thus requiring clinician review. However, we believe this is a reasonable compromise to ensure clinical safety within an automated process. Moreover, the process of review is fast in case of data falsely labeled as erroneous, which only requires a visual check from the clinician to accept the analysis results.

### Full Pipeline: From Scanner to Report

Using the image-processing steps developed in this paper, we were able to present the first pipeline for analysis of cardiac function from cine CMR that automates the complete process from scanner to report, offering an automated system that reproduces manual analysis in current clinical practice. This pipeline is characterized by a high degree of QC (one step in the new framework, two steps in the previously published one). Sequential QC steps focusing on different quality problems ensures a “Swiss cheese” framework, where if a poor-quality image slips through a first barrier, it will likely be flagged up in a later stage. Supplementary Video 1 displays how the new quality control step aids in selecting images where segmentation can be performed at high standards by subsequent pipeline steps.

Moreover, the addition of the new framework offers automated selection of all standard cine views, which can be further exploited for analysis of parameters beyond conventional ones, such as longitudinal strain and atrioventricular valve systolic excursion, expanding the role of CMR for the assessment of systolic and diastolic function.

The full pipeline is highly accurate, with a focus on high sensitivity, showing an improvement compared to our previously published work (5). The pipeline is also significantly time-efficient, producing outcome measures in about 4 min for standard scans, and in up to 7 min for longer research scans. This is faster than the time reported for the initial pipeline (5), due to changes in the previously developed code.

In the future, we aim to extend our framework to identify and analyze other CMR sequences, including late gadolinium enhancement, flow and T2 mapping. Moreover, with an expanding data set we will be able to train quality control CNNs to recognize specific types of quality and planning errors. In order to decrease subjectivity of this task, a collaborative initiative to build a consensus across a vast number of operators, similar to a recent one developed in the field of echo and AI (25), would be of great value. Lastly, our method now requires post-processing and is separated from the CMR scanner. In the future, effort should be made for direct implementation on the scanner console. In particular, the implementation of CNN_QC_ at the time of image acquisition would aid radiographers by promptly detecting images of unsatisfactory quality. This would improve image quality upstream and yield a greater accuracy of downstream image analysis (26).

### Study Limitations

Our dataset did not include CMR studies acquired with General Electrics (GE), thus limiting our framework's generalizability. However, using Philips and Siemens granted a high degree of variability, which would facilitate further training with GE data.

DL algorithms inherently are black boxes. Therefore, interpretation of decisions remains challenging. In this paper we used a stepwise approach of classification and QC algorithms instead of a fused algorithm to allow at least some interpretation of the DL-based decisions.

Moreover, although we included a large number of patients with ACHD to train and test the model, exclusion of grossly distorted anatomy limits the use of this framework in patients with severe ACHD.

The framework presented in this paper performs limited quality control for short axis. We decided not to train a further CNN for this view as already present in the previously validated pipeline, and it would have therefore been redundant.

## Conclusions

We developed and validated a framework to select cine acquisitions and perform QC of the selected images prior to automated cine CMR image analysis. We show that our network is able to select cine CMR from a full clinical CMR exam accurately and screen for image quality with a high rate of detecting erroneous acquisitions. We implemented our developed framework as the first step of a wider quality-controlled pipeline to obtain automated, quality-controlled analysis of cardiac function from short and long axis cine images from complete CMR clinical studies.

## Data Availability Statement

The datasets presented in this study can be found in online repositories. The names of the repository/repositories and accession number(s) can be found below: The UK Biobank data set is publicly available for approved research projects from https://www.ukbiobank.ac.uk/. The GSTFT data set cannot be made publicly available due to restricted access under hospital ethics and because informed consent from participants did not cover public deposition of data.

## Ethics Statement

The studies involving human participants were reviewed and approved by the North West Research Ethics Committee (REC 11/NW/0382; for UK Biobank data) and the South London Research Ethics Committee (REC 15/NS/0030; for GSTFT data) and was conducted according to the Declaration of Helsinki and Good Clinical Practice guidelines. The patients/participants provided their written informed consent to participate in this study.

## Author Contributions

VV, EP-A, and BR: conception, study design, and data analysis. EP-A: development of algorithms and analysis software. VV and EP-A: data pre-processing. VV, BR, and RR: clinical advice. VV and BR: manual image annotation. EP-A, BR, and RR: interpretation of data and results. VV, EP-A, BR, and RR: drafting and revising. All authors have read and approved the final manuscript.

## Funding

This work was supported by the Wellcome/EPSRC Center for Medical Engineering at Kings College London (WT203148/Z/16/Z). EP-A was supported by the EPSRC (EP/R005516/1 and EP/P001009/1) and by core funding from the Wellcome/EPSRC Center for Medical Engineering (WT203148/Z/16/Z) and BR was supported by the NIHR Cardiovascular MedTech Co-operative award to the Guy's and St. Thomas' NHS Foundation Trust. This research was funded in whole, or in part, by the Wellcome Trust WT203148/Z/16/Z. For the purpose of open access, the author has applied a CC BY public copyright license to any author accepted manuscript version arising from this submission. The authors acknowledge financial support from the Department of Health through the National Institute for Health Research (NIHR) comprehensive Biomedical Research Center award to Guy's and St. Thomas' NHS Foundation Trust in partnership with King's College London.

## Author Disclaimer

The views expressed are those of the authors and not necessarily those of the NHS, the NIHR, EPSRC, or the Department of Health.

## Conflict of Interest

The authors declare that the research was conducted in the absence of any commercial or financial relationships that could be construed as a potential conflict of interest.

## Publisher's Note

All claims expressed in this article are solely those of the authors and do not necessarily represent those of their affiliated organizations, or those of the publisher, the editors and the reviewers. Any product that may be evaluated in this article, or claim that may be made by its manufacturer, is not guaranteed or endorsed by the publisher.

## Supplementary Material

Supplementary Video 1 Full pipeline: from scanner to report. It displays the fully automated multi-step QC pipeline from scanner to image analysis and how implementing the new framework prior to segmentation results in good quality analysis.

## Abbreviations

2Ch: 2-Chamber view
3Ch: 3-Chamber view
4Ch: 4-Chamber view
ACHD: adult congenital heart disease
AI: artificial intelligence
CMR: cardiac magnetic resonance
CNN: convolutional neural network
DL: deep learning
GE: General Electrics
GSTFT: Guy's and St. Thomas’ NHS Foundation Trust
LVOT: left ventricular outflow tract
QC: quality control
SAX: short axis.
